# “Spazio Huntington”: Tracing the Early Motor, Cognitive and Behavioral Profiles of Kids with Proven Pediatric Huntington Disease and Expanded Mutations > 80 CAG Repeats

**DOI:** 10.3390/jpm12010120

**Published:** 2022-01-17

**Authors:** Federica Graziola, Sabrina Maffi, Melissa Grasso, Giacomo Garone, Simone Migliore, Eugenia Scaricamazza, Consuelo Ceccarelli, Melissa Casella, Ludovica Busi, Barbara D’Alessio, Alessandro De Luca, Giovanna Stefania Colafati, Umberto Sabatini, Alessandro Capuano, Ferdinando Squitieri

**Affiliations:** 1 Neurology Unit, Department of Neurosciences, IRCCS Bambino Gesù Children Hospital, 00146 Rome, Italy; federica.graziola@opbg.net (F.G.); melissa.grasso@opbg.net (M.G.); giacomo.garone@opbg.net (G.G.); alessandro.capuano@opbg.net (A.C.); 2 Italian League for Research on Huntington Foundation, Via Varese 31, 00185 Rome, Italy; consuelo.ceccarelli@lirh.it (C.C.); melissa.casella@lirh.it (M.C.); ludovica.busi@lirh.it (L.B.); barbara.dalessio@lirh.it (B.D.); 3 Huntington and Rare Diseases Unit, Fondazione IRCCS Casa Sollievo della Sofferenza Hospital, Viale Cappuccini, 71013 San Giovanni Rotondo, Italy; sabrina.maffi@gmail.com (S.M.); sim.migliore@gmail.com (S.M.); eugenia138@hotmail.com (E.S.); 4 Medical Genetics Division, Fondazione IRCCS Casa Sollievo della Sofferenza Hospital, Viale Cappuccini, 71013 San Giovanni Rotondo, Italy; a.deluca@css-mendel.it; 5 Neuroradiology Unit, Imaging Department, IRCCS Bambino Gesù Children Hospital, 00146 Rome, Italy; gstefania.colafati@opbg.net; 6 Neuroradiology Unit, Department of Medical and Surgical Sciences, Magna Graecia University, Viale Europa, 88100 Catanzaro, Italy; sabatini@unicz.it

**Keywords:** pediatric Huntington disease, observational studies, prospective studies, high CAG expansions, atypical Huntington disease

## Abstract

The “Spazio Huntington—A Place for Children” program was launched in 2019. The aim was to contact at risk kids within Huntington disease (HD) families, to provide counseling to their parents and to start a prospective follow-up of kids suspicious to manifest pediatric HD (PHD). We met 25 at risk kids in two years, four of whom with PHD and highly expanded (HE) mutations beyond 80 CAG repeats. We rated motor, neuropsychological and behavioral changes in all PHD kids by the Unified HD Rating Scale (UHDRS)-total motor score (TMS) and additional measures of (1) cognitive level (Leiter International Performance Scale), (2) adaptive functioning (Adaptive Behavior Assessment Systems), (3) receptive language (Peabody Picture Vocabulary Test) and (4) behavioral abnormalities (Child Behavior Check List and Children’s Yale–Brown Obsessive Compulsive Scale). All PHD kids showed a severe progression of neurological and psychiatric manifestations including motor, cognitive and behavioral changes. The magnetic resonance imaging contributed to confirm the suspicious clinical observation by highlighting very initial striatum abnormalities in PHD. Spazio Huntington is a program to prospectively study PHD, the most atypical face of HD, and may represent the basis to recruit PHD patients in future clinical trials.

## 1. Introduction

Huntington disease (HD) is an autosomal dominant neurodegenerative disorder, caused by an expansion of an unstable trinucleotide CAG repeat in the exon 1 of *HTT* gene, which translates into an elongated polyglutamine stretch in Huntingtin protein [1]. The larger the CAG repeat expansion, the earlier the age at onset, with childhood onset associated with mutations of 60 repeats and more [2], even though other factors affect the mutation penetrance [3].

HD is characterized by neurological, behavioral, and cognitive changes. Onset symptoms may start at any age, even though they generally occur in adulthood around 40 years of age [4]. Involuntary movements, i.e., chorea, do represent a typical onset manifestation in the adult patients, even though the atypical occurrence of onset symptoms may sometime predominate over choreic movements since the beginning of HD [5]. When HD starts before 21 years of age, it is conventionally defined as the juvenile-onset HD (JOHD). Among JOHD patients, the minors who manifest HD determine the pediatric variant (Pediatric HD or PHD). Approximately 5–10% of all HD cases may manifest with JOHD [6], while the frequency of affected kids, who meet the definition of PHD, is still undefined [7].

More recently, a study revealed that the variant including pediatric-onset kids with highly expanded (HE) mutations beyond 80 CAG repeats showed an aggressive disease progression with reduced life span, motor and psychic developmental delay, early gait disturbance, severely progressive neurological impairment with progressive dystonia, no chorea like in adults, poor school performance, seizures and an atypical pattern of brain abnormalities [8]. Behavioral disturbances like hyperactivity and oppositional and aggressive behavior are also frequently reported in JOHD and PHD cohorts [2,8,9].

To date, only few observational retrospective reports described changes in cognition and behavior in JOHD cohorts on the basis of qualitative and caregivers’, self-reported, questionnaires. These qualitative descriptions included heterogeneous cohorts of patients, selected according to unspecific clinical presentations, starting with either neurological or behavioral symptoms [10,11,12,13,14,15]. Most of these studies missed any preventive biological stratification based on the CAG mutation size in the *HTT* gene [16]. Currently, data delineating the early development of motor, cognitive and behavioral symptoms in childhood-onset patients with large expansions are still missing [17]. This is due to the difficulty in observing the rare and HE children since the very beginning of their pathological condition because they develop symptoms very early in life and progress very fast. Thus, the dramatic worsening of their condition severely limits the possibility to define the profile of early clinical changes, mainly in cognition.

In this preliminary observational study we aimed at tracing the first clinical modifications in a cohort of PHD kids, who had a genetic confirmation of large sized CAG mutations associated with very early onset of motor, cognitive and behavioral onset manifestations.

## 2. Materials and Methods

As a first step, we reviewed all pedigrees collected by direct interviews to HD families from all over Italy, whose data have been entered into the institutional database held by the Italian League for Research on Huntington Foundation, Rome, Italy (start date March 2001, still active) [8,18]. Such an analysis offered the opportunity to preliminarily select those families with kids at risk of HD, whose parents had expressed the wish to allow their participation in observational programs.

In July 2019, in partnership with Bambino Gesù and Casa Sollievo della Sofferenza Research Hospitals, the Italian League for Research on Huntington (LIRH) Foundation launched the “Spazio Huntington—A Place for Children” program, with the intent to increase the parents’ awareness on at risk kids who may potentially show suspicious clinical manifestations of HD. Spazio Huntington offers families the opportunity to meet a multidisciplinary team of specialists (e.g., neurologists, pediatricians and psychologists) in a nonmedical environment located at LIRH Foundation headquarters in Rome. According to the International Guidelines for Genetic Testing in Minors [19], we performed the genetic test only in those children who showed suspicious clinical features, after the parents’ signed informed consent. By ‘suspicious clinical features’ we mean clinical manifestations including neurological abnormalities, with or without cognitive and/or behavioral changes, associated with a motor impairment scored by the Unified Huntington’s Disease Rating Scale—total motor score (UHDRS-TMS) ≥10 and diagnostic confidence level (DCL) = 4 [8,20].

Once we observed suspicious clinical features, we counselled the parents [21] and, with their consent, we started a specific clinical kids’ screening. The age at onset, medical history and any other relevant event were investigated by interviewing both parents, according to the published procedure which took into account all major motor and cognitive abnormalities that the child started to manifest since the birth [8,11]. Cognitive and behavioral assessments were performed at the Neurology Unit of the Pediatric Bambino Gesù Research Hospital in Rome. The neuropsychological testing battery included the measure of the cognitive level, adaptive functioning, receptive language. Specifically, all patients underwent the following standardized tests: Intelligence level was assessed using Leiter International Performance Scale [22] (M: 100, SD: 15) a non-verbal cognitive ability test suitable for individuals with speech problems. Adaptive skills were assessed by administering to the patients’ caregivers the Adaptive Behavior Assessment Systems (M:100, SD:15), a standardized questionnaire assessing adaptive functioning in the conceptual, social, and practical domain [23]. Receptive vocabulary was evaluated using the Italian version of Peabody Picture Vocabulary Test [24] (M: 100, SD: 15). Target words were orally presented, and participants responded by pointing at the target-picture choosing among four images. Behavioral problems were evaluated through a parent’s questionnaire, the Child Behavior Check List (CBCL) [25], and a doctor semi-structured interview, the Kiddie Schedule for Affective Disorders and Schizophrenia [26]. Moreover, obsessions and compulsions were screened using the Children’s Yale–Brown Obsessive Compulsive Scale (CY-BOCS) a self-report tool used to aid in the assessment of obsessive–compulsive symptoms and diagnosis of obsessive–compulsive disorder in children [27]. The rating scale consists of 10 questions and gives a CY-BOCS total score ranging from 0 to 40 [27].

A specific molecular genetic test for HD children to detect mutations with high CAG repeat number was performed at CSS-Mendel Institute in Rome, branch of IRCCS Casa Sollievo della Sofferenza Research Hospital [28].

All HE patients performed a brain magnetic resonance imaging (MRI) in the same instrument machine at the IRCCS Bambino Gesù Hospital. All images were generated from a 3.0 Tesla MRI scanner (Siemens Magnetom Skyra, Erlangen, Germany) and the imaging protocol included with T2-weighted, FLAIR (fluid-attenuated inversion recovery), diffusion-weighted imaging and 3D T1-weighted MPRAGE (magnetization prepared rapid gradient echo imaging) sequences. The current analysis refers to a medical assay for diagnostic purposes only.

The program was ethically approved, and informed consent was obtained from all subjects’ parents involved in the study. The current study refers to the clinical observation of four kids, who were met in the context of the “Spazio Huntington—A Place for Children” program, between July 2019 and February 2021 and entered a prospective study after the clinical diagnosis of PHD. Retrospective data concerning the UHDRS-TMS were also collected for longitudinal motor tracking of the disease. All kids were examined by the same team operators from LIRH Foundation, CSS-Mendel/IRCCS Casa Sollievo della Sofferenza and Bambino Gesù Research Hospitals, with expertise of HD in children.

## 3. Results

### 3.1. Clinical Overview

**Figure 1:**
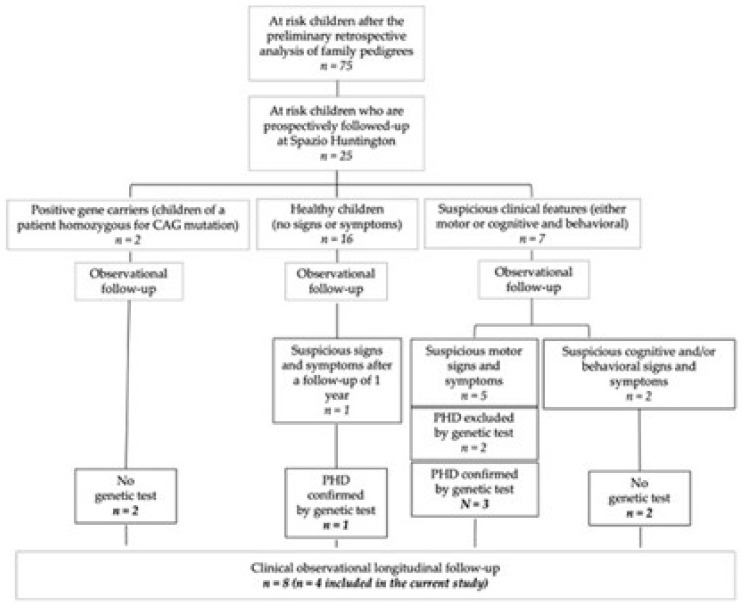
Stratification of Spazio Huntington cohort. Flow chart showing the strategy of selection and stratification of the cohort, starting from a preliminary datasheet analysis of pedigrees and further interviews to families which were then met at “Spazio Huntington—A Place for Children”. The genetic test was only provided after a suspicious neurological exam highlighted a diagnosis which was suggestive of PHD (pediatric HD). All the confirmed PHD cases had a brain magnetic resonance imaging consistent with the diagnosis of PHD, according to the documented evidence, as described in [12]. In some cases the follow-up of children of expanded CAG homozygous patients may offer an opportunity to prospectively follow-up young subjects many years in advance before onset manifestations.

**Figure 2:**
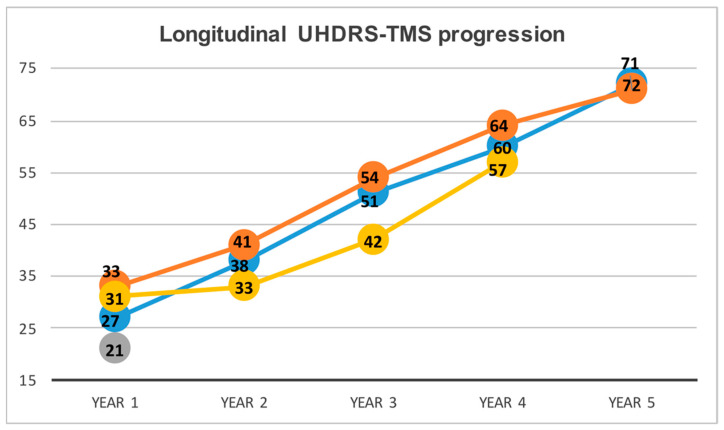
Longitudinal UHDRS-TMS progression. UHDRS: Unified Huntington’s Disease Rating Scale. TMS: total motor score. Each line refers to a single patient. Three out of four patients showed a remarkable yearly TMS change indicating a severe motor progression of the disease. TMS is reported within each circle. Orange = Patient 1, yellow = Patient 2, blue = Patient 4 and grey = Patient 3, the youngest patient, with only a basal assessment was available.

The preliminary screening of HD pedigrees highlighted a cohort of 75 children (i.e., with at least one parent who discovered to be mutation carrier) (Figure 1). In two years, we had the opportunity to meet at-risk children and their parents once a month, with the obvious limitations due to COVID-19 disease due to SARS-CoV-2 Coronavirus-2 restrictions occurred in 2019 and in 2020. In total, we met 25 kids and their parents. The Figure 1 shows a flow-chart that summarizes the whole cohort stratification. Of the 25 kids, four (two females) carrying highly expanded mutations included between 84 to 114 CAG repeats inherited by the affected father, manifested with PHD (Table 1). The childhood age of onset was preschooler and ranged between age 2 and 4 years. The age of diagnosis ranged between 5 and 7 years with a mean diagnostic delay of 3 years (Table 1). Clinical details, including first developmental steps, cognitive assays and main neurological features with UHDRS-TMS changes, were reported in Table 2, Table 3 and Table 4, respectively. The yearly longitudinal TMS profile is reported in Figure 2. The medical history revealed that all patients’ mothers had a regular delivery, and all patients had a regular perinatal period. Two of their mothers had an uneventful pregnancy (Patient 1 and 2, Table 2), while the other two had gestational diabetes and a transitory pregnancy hypertension in one case (Patient 3, Table 2) and transitory pregnancy hypertension in the other case (Patient 4, Table 2). The two girls had a medical history of developmental delay, while the two boys had apparently a regular psychic and motor development. Three out of four patients showed a mixed sleep disorder with frequent awakenings and difficulty in falling asleep with onset at the time of the diagnosis. They all reported drooling after the onset of motor symptoms and three of them developed progressive feeding difficulties in the following years (Table 2). Three patients (Patient 1, 2 and 3) underwent a neuropsychological testing battery to measure cognitive level, adaptive functioning and receptive language. We found a mild intellectual disability ranging from 68 to 73 (Intelligence Quotient (IQ) M: 100, DS: 15), a receptive language delay and a poor adaptive functioning in all tested patients (Table 3). We also conducted a behavioral evaluation through a semi-structured interview and parent report from which we disclosed a behavioral problem in three of four patients (Patients 1, 2 and 4) characterized by low impulsivity control and explosive outbursts of anger with disproportionate reaction to any provocation (Table 3). Moreover, it was reported an avoidant/restrictive food intake disorder in one patient (Patient 1), attention deficit disorders in another patient (Patient 2) and social isolation in a patient (Patient 4).

Among the most common features which appeared within the first years of life there were the unstable walking with propensity to falls and difficulty in the speech production and language acquisition (Table 3).

The TMS increased during the prospective follow-up in three out of four kids with a consistent gain of scale units from Year 1 to Year 3 (i.e., range included between 26 and 45 increase of units) (Table 4). The final TMS was consistent with a severe clinical condition (e.g., 57, 71 and 72 TMS units), which is usually observed in the advanced adult-HD (Table 4 and Figure 2). Three out of four patients manifested epilepsy with generalized tonic-clonic seizures between 4 to 7 years after the onset (Table 4).

**Figure 3:**
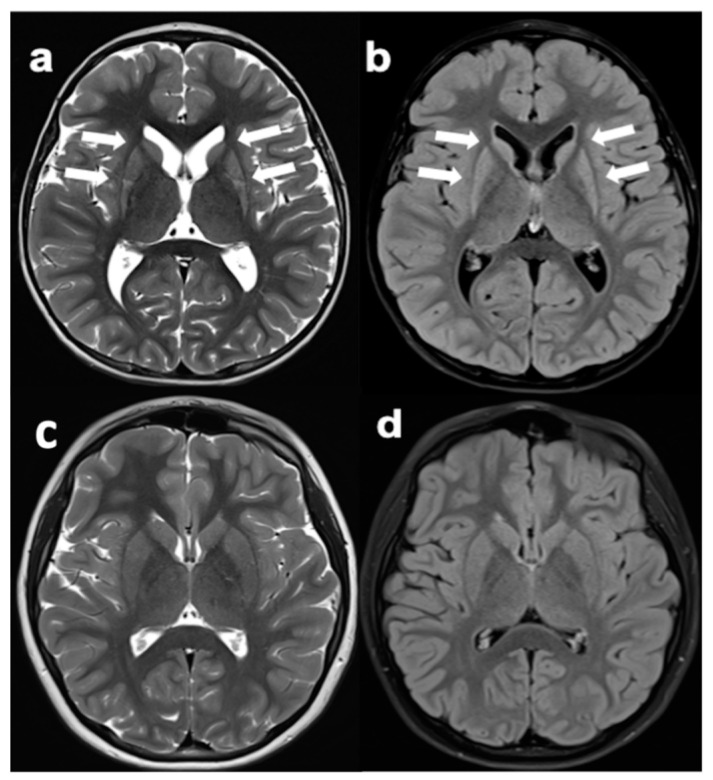
MRI (Magnetic Resonance Imaging) in a HE-PHD (highly expanded- pediatric Huntington disease) patient (Patient 3) and in a subject with suspicious motor manifestations of PHD. (**a**,**b**) Patient 3 with a pathological total motor score = 21 performed MRI at the very beginning of PHD (MRI age = 5, age at onset = 3.5). Atrophy and slight signal hyperintensity of the caudate nuclei and putamina (arrows). (**c**,**d**) At risk kid with total motor score = 20 and suspicious PHD. Normal appearance of basal ganglia, with no signs of atrophy or signal alterations.

MRI showed a remarkable volume loss and altered signal of the basal ganglia structures, particularly the striatum, in all HE patients (Figure 3).

### 3.2. Longitudinal Clinical History

#### 3.2.1. Case 1

This was a 10 year old girl with a positive HD family history and a 95 CAG repeat mutation (Table 1). Her affected father refused to perform the genetic test. She manifested the disease with a developmental delay, i.e., autonomous ambulation acquired at about 18–19 months of age but never described as stable and first words at 24–30 months followed by a slow acquisition of both receptive language and speech production. At age 4, she started to present a clear gait disorder with frequent falls. At age 6 she underwent a formal verbal psychometric assessment (WISC: IV—Wechsler Intelligence Scale for Children 4th edition) disclosing a low IQ (71) and a diagnosis of mild intellectual disability was set. At age 9 the assessment was repeated with a non-verbal psychometric assessment (LEITER-3) confirming the mild intellectual disability (IQ: 70). She increased her UHDRS-TMS from 27 to 45 units in three years. She rapidly developed bradykinesia with dystonia, dysarthria, and behavioral problems including explosive outbursts with poor impulsivity control. She developed an avoidant/restrictive food intake disorder with associated obsessive thoughts with poor insight. She also presented drooling and swallowing difficulties and a sleep disturbance of initiating sleep and frequent awakenings. She developed a compulsive behavior with caregiver’s dependence and co-sleeping. At 9.5 years old, she started to manifest apraxia and generalized epilepsy with tonic-clonic seizures. A treatment with valproic acid and levetiracetam was started with discrete control of seizures.

#### 3.2.2. Case 2

A 12 year old boy with a positive HD family history and an 87 CAG repeat mutation inherited from the affected father (Table 1). He manifested onset symptoms at 4 years of age with frequent falls and gait impairments. Few years later he manifested a speech disorder with dysarthria. At 8 years, after the diagnosis, he manifested generalized seizures and started a treatment with valproic acid. Motor symptoms rapidly deteriorated with worsening of walking, bradykinesia and dystonia. He increased his UHDRS-TMS from 41 to 71 units in three-year follow-up. He also developed drooling, swallowing difficulties, stereotypic manifestations and apraxia at age 10. He underwent a non-verbal psychometric assessment (LEITER-3) revealing a mild intellectual disability (IQ: 68) associated with severe impairment in the adaptive functioning (IQ: 34). From a behavioral perspective, he presented explosive outbursts with impulsivity, attention deficit disorder and sleep disturbance of initiating sleep and frequent awakenings.

#### 3.2.3. Case 3

A 4.5 year old boy with a positive HD family history and a 114 CAG repeat mutation inherited from the affected father (Table 1). We met this child at the very beginning of his clinical manifestations, occurring at age 3.5 with slight dystonic postures and bradykinesia, slightly affecting his gait. He underwent a psychometric assessment (LEITER-3) revealing a mild intellectual disability (IQ: 73) associated with social isolation and initial explosive outbursts. In depth neurological assessment, including motor assay and TMS were unavailable due to the oppositional child’s behavior.

#### 3.2.4. Case 4

A 11-year-old girl with a positive HD family history and an 84 CAG repeat mutation inherited from the affected father (Table 1). She presented a developmental delay both in motor and language area. She started to walk at 18–20 months of age and her first words started at about 2 years of age, with a language disorder both for receptive and expressive language. With the beginning of elementary school, she started to present learning difficulties and a worsening of motor abilities with frequent falls and refuse of autonomous walking. She soon developed a bradykinetic dystonic syndrome. She increased her UHDRS-TMS from 31 to 57 units in three years and developed drooling and swallowing difficulties since age 10. She also developed behavioral problems, particularly explosive outbursts with hetero- and auto-aggressive behavior, low impulsivity control and social isolation. She presented obsessions and compulsions with excessive food intake and a sleep disturbance of initiating sleep and frequent awakenings and co-sleeping. Unfortunately, her clinical conditions did not allow a reliable neurocognitive assessment.

## 4. Discussion

HD is typically an adulthood illness, rarely starting in minors and exceptionally beginning within the first decade of life [8,15,17,29]. PHD kids are considered ultra-rare and there has never been an epidemiological estimation of their frequency so far. Such a condition is particularly severe, largely underestimated due to its atypical presentation, which does not allow timely diagnoses, and causes a huge discomfort within HD families [15,30]. One of the greatest discomforts is due to the parents’ unacceptance of the offspring’s clinical conditions because of the preschool and primary school stigma around their children. Another important discomfort is the parents’ fear that their children may face a severely progressive and relentless disease [30]. Moreover, the delay in the clinical diagnosis represents a strong limitation in delineating the natural history in these cases.

Such a dramatic condition induced us to promote a strategy to approach families in an as protected environment as possible. Indeed, our program named “Spazio Huntington—A Place for Children”, aims to approach families with at risk kids by meeting them in a nonmedical environment, where they may meet other kids with limited or no risk to cross adult patients, as it may happen in an ‘ordinary’ clinic. Our strategy, aiming to regularly meet and observe minors, may allow us to prospectively follow-up kids, since before they show signs and symptoms of HD, like we do with at risk adults, in the full respect of ethical guidelines. Thanks to such a program, we had the chance to see PHD kids at the beginning of their disease.

We here describe a cohort of four patients with PHD since their first disease phases and followed up across years after the first HD manifestations. Despite the limited size, we think our cohort is of great relevance considering the rarity of such a young population which we stratified according to several specific clinical and genetic characteristics and considering the important potentiality to allow prospective follow-ups, since the beginning of the disease. Furthermore, our approach consents the clinical observation before the full development of features such as bradykinesia or severe dystonia, that become visible in fully manifest kids, sometime several years after the disease onset [8,15].

So far, all studies concerning cognitive and behavioral changes in PHD cohorts have been performed by caregiver-based, self-reported, retrospective descriptions, with no focus on specific and comprehensive evaluations [8,9,16] or by caregiver survey [14,31]. Few studies reported a retrospective analysis in JOHD with neuropsychological examinations only performed in young adults (i.e., aged 20 years) and almost never in affected kids, generally showing limited length mutations [15,16]. Therefore, our study, despite the limitation due to the cohort size, represent an exhaustive analysis of a quantitatively assessed motor impairment (i.e., TMS) and objectively measured behavioral and cognitive changes by an extensive battery of neuropsychological tests in kids with PHD and large CAG mutations, the most atypical, rare, and severe HD face.

Among the peculiarities we highlight in our study, we describe and confirm the high occurrence of sleep disorders in affected young children, in line with previous reports on JOHD [14]. We also confirm the occurrence of seizures as a common feature in PHD [8,31,32,33]. Furthermore, we report the occurrence of drooling, dysphagia, stereotypic movements and apraxia during the first phases of the disease, all features that may concur to worsen the disease severity and shorten the patients’ life span, as described [8]. Our clinical study may provide the opportunity to detect peculiar clinical changes at the very beginning of the kids’ life, by tracing the first infant’s developmental steps. It also raises the interesting question of whether we have been really able to detect all clinical manifestations in affected HE kids so far or should we instead revise the clinical approach to correctly assay the age at onset in kids.

The patients who performed a structured cognitive assessment showed a mild intellectual disability in the context of a language delay and a poor adaptive functioning. The two young girls presented a history of developmental delay both for language and for motor areas (i.e., delay in gait initiation), while the two young boys apparently manifested by regular developmental milestones in the first year of life. Due to the limited size of our cohort, we cannot raise conclusions concerning the effect of the gender and of the mutation size on the developmental delay in HD. Larger numbers are needed to confirm our observation. However, one of the two boys, we had the opportunity to follow-up since the beginning of his clinical manifestations at age 3.5 and who showed the largest repeat expansion of 114 CAG, was objectively tracked since the very beginning of his clinical condition and showed very early, even though initial, cognitive changes. Such a prospective tracking of PHD does certainly represent an exceptional opportunity to highlight changes that are as close to premanifest life stage as possible in affected kids’ life. We believe this strategy could therefore represent the best way to disclose the natural history of PHD. Our approach opens to the possibility to prospectively follow-up young subjects, years in advance before disease manifestations. We have to remember that this strategy has been successful to detect potential biomarkers in adults [34].

Our observation highlighting that some children show a normal developmental milestone acquisition, even though limited to the first years of life, despite they carry HE mutations, raises also the question of whether PHD is entirely a neurodevelopmental delay illness or, instead, the abnormal neurodevelopment just represents a contributor to biologically speed up neurodysfunctional and neurodegenerative processes, further occurring during life. For instance, our findings need to be contextualized within the current HD scenario. For example, we and others reported the evidence of features of developmental delay in affected young girls and boys with large expansions [8,10,12]. The neurodevelopmental delay was retrospectively documented in two boys with very large mutations and age at onset of 18 months [12,29], one of them with very highly expanded CAG of about 200 repeats [29]. Therefore, we need to take into account the several biological (e.g., mutation length, genetic background, gene modifiers) and the yet unknown environmental (e.g., family environment, pre-delivery stressors, etc.) factors, that may theoretically interfere with the normal brain development and with the biological disease processes. Additional imaging and neuropathological studies in PHD patients are strongly needed to answer to the many, yet unsolved, questions concerning the contribution of developmental and degenerative processes to the timing of clinical disease presentation and progression [17].

Another interesting difference between affected girls and boys consisted in how the behavioral changes developed. While explosive outbursts and low impulsivity control were common factors of all the patients, no obsessive-compulsive disorders symptoms were reported by the two boys, while the two girls reported a frequent occurrence of obsessions and compulsions. In these two cases, the compulsions were mainly focused on alimentary behavior with one girl showing an avoidant/restrictive food intake disorder and the other one bulimic-like attacks.

We here describe a preliminary clinical analysis of PHD kids who are followed up at “Spazio Huntington—A Place for Children”, a HD family-centered initiative, with the final aim to contribute to clinical observational research, also by raising awareness on a rare, severe, variant of a rare, severe, disease. Although our results should be seen in light of the small size of our cohort and of the exploratory nature of our study, we believe our approach may help to detect PHD cases at the very beginning, thus representing an example of strategy to recruit children for further clinical trials. Our approach may also offer the opportunity for prospective and long-term evaluations which are needed to collect biological and reliable clinical data for research on HD mechanisms and biomarkers.

Our study confirms our previous retrospective data where we documented that the disease course in HD kids with HE mutations was more severe than adult-onset patients [8]. The prospective follow-up of our HD kids in the current study shows a severe neurological progression with particularly large increase of UHDRS-TMS units compared to what is expected in adult-onset patients [20]. Furthermore, the test we used to assay neuropsychological and behavioral changes may represent an important start point for further prospective analyses in this cohorts and might offer new tools for tracking the disease in children. Of note, all the four kids had the previously described brain imaging pattern, i.e., early abnormal loss of striatum volume [8]. Interestingly, the children who showed a suspicious clinical picture (with a pathological TMS), but resulted negative at the genetic test, did not show the same consistent pattern. Our observation highlights the brain MRI potentiality to ethically address a correct clinical and genetic screening in minors who are at risk of HD and show subtle symptoms. Our findings lend support to the hypothesis that the TMS has important implication in PHD kids by tracking the disease progression, while the volumetric brain MRI may have a critical implication as an additional, specific, diagnostic tool. We hope to contribute to elucidate the important role of imaging in PHD by exploring the brain function and metabolism, in addition to the brain structure, longitudinally by our in progress RAREST-JHD project.

Whether a longitudinal MRI analysis, to extend to other brain regions (e.g., cerebellum, brain cortex, brainstem, etc.), may help in monitoring the HD progression to trace the brain development of HD in kids, still needs in depths imaging and neuropathological studies including structural, functional, and metabolic studies [35].

Finally, the careful analysis of the first developmental steps, retrospectively collected by interviewing kids and kids’ parents in a nonmedical environment such as “Spazio Huntington—A Place for Children”, highlighted previously undetected clinical features in both mothers (e.g., gestational diabetes, pregnancy hypertension) and children (e.g., weight at birth, development timing). Such features may contribute to enrich a dedicated data bank for potential use and correlation with clinics in future, thus contributing to update other large dataset collection such as the Enroll-HD platform.

Kids’ recruitment in observational and therapeutic trials does represent a relevant challenge for researchers. In addition, there is more: it is also an important regulatory agencies recommendation to extend clinical trials protocols to minors who manifest symptoms of HD [36]. Considering the shorter life span of HD kids compared with the adult’s [8], prospective studies are a pre-requisite to make HD affected kids’ recruitment into therapeutic trials really possible. “Spazio Huntington—A Place for Children”, can be seen as the Italian contribution to achieve such an important goal, for the benefit of the whole HD global community.

## Figures and Tables

**Table 1:** Demographics.

Patient No.	Patient Code	CAG Exp.	Gender M/F	Genetic Transmission	Age at Onset (Years)	Age at Diagnosis (Years)
1	HD130 05	95	F	paternal	2	6
2	HD379 05	87	M	paternal	4	7
3	HD707 02	114	M	paternal	3.5	5
4	HD636 01	84	F	paternal	2	6

The patient code anonymizes the collection of patient’s data and samples. Exp.: expansions; M/F: masculine/feminine; HD: Huntington disease.

**Table 2:** First developmental steps.

Patient No.	Patient Code	Gender	CAG Exp.	Major Mother’s Pregnancy Events	Weight at Birth (gr)	Walking (First Independent Steps)	Balance (Tendency to Fall)	Speech Production (First Words)	Language Acquisition
1	HD130 05	F	95	Birth at term	3000	18–19 months	Unstable	24–30 months	Blurred speech
2	HD379 05	M	87	Birth at term	3550	12 months	Unstable	10–12 months	Stuttering
3	HD707 02	M	114	Birth at term, gestational diabetes and pregnancy hypertension	3670	12 months	Ok	12 months	Fluid language
4	HD636 01	F	84	Birth at term, gestational diabetes	3640	20–22 months	Falls	18 months	Never fluid

All patients inherited the highly expanded (HE) mutation from their affected father. Language was never fluid in females (No. 1 and 4). Patient 2 showed intermittent/involuntary repetitions of syllables. Exp.: expansions; HD: Huntington disease; M: masculine; F: feminine.

**Table 3:** Cognitive and behavioral assay.

Patient No.	Patient Code	Age at Evaluation (Years)	LEITER Cognitive Level	ABAS Adaptive Functioning	PPVT Receptive Language	CY-BOCS Obsession and Compulsions	CBCL Behavior Problems—Parent(s) Report	K-SADS Behavior Problems—Health Professional’s Interview
1	HD130 05	10	IQ = 70	IQ = 63	IQ = 76	22/40	Anxiety and affective problems	Explosive outbursts, low impulsivity control, avoidant/restrictive food intake disorder
2	HD379 05	12	IQ = 68	IQ = 34	IQ < 65	0/40	Social and affective problems	Explosive outbursts, low impulsivity control and attention deficit disorder
3	HD707 02	4	IQ = 73	IQ = 68	IQ = 70	0/40	No	Explosive outburst and social isolation
4	HD636 01	11	NA	NA	NA	20/40	Anxiety, social and affective problems	Learning difficulties, explosive outbursts with hetero and auto-aggressive behavior, low impulsivity control and social isolation

IQ: intelligence quotient; NA: not available; LEITER: Leiter International Performance Scale; ABAS: Adaptive Behavior Assessment Systems; PPVT: Peabody Picture Vocabulary Test; CY-BOCS: Children’s Yale–Brown Obsessive Compulsive Scale; CBCL: Child Behavior Check List; K-SADS: Kiddie Schedule for Affective Disorders and Schizophrenia.

**Table 4:** Main neurological features.

Patient No.	Patient Code	Age at the First Assay in Years (Age at Onset)	TMS at the First Assay	Age at the Last Assay (Years)	TMS at the Last Assay	Gain of TMS Units from the First to the Last Assay	Total Motor Score Units/Year from Onset Age	Age of First Epileptic Seizure (Years)
1	HD130 05	7 (2)	27	10	72	+45	9	9
2	HD379 05	9 (4)	41	13	71	+30	7.8	8
3	HD707 02	4 (3.5)	NA	5	NA	NA	NA	No
4	HD636 01	8 (2)	31	11	57	+26	6.3	13

TMS: total motor score; NA: not available.

## Data Availability

The data that support the findings of this study are available from the corresponding author upon reasonable request.
